# Transcatheter Closure of Mitral Paravalvular Leak via Multiple Approaches

**DOI:** 10.1155/2021/6630774

**Published:** 2021-03-02

**Authors:** Yang Liu, Chennian Xu, Peng Ding, Jiayou Tang, Ping Jin, Lanlan Li, Min Chen, Xin Meng, Hongliang Zhao, Jian Yang

**Affiliations:** ^1^Department of Cardiovascular Surgery, Xijing Hospital, Air Force Medical University, Xi'an, China; ^2^Department of Anesthesiology, Xijing Hospital, Air Force Medical University, Xi'an, China; ^3^Department of Ultrasound Medicine, Xijing Hospital, Air Force Medical University, Xi'an, China; ^4^Department of Radiology, Xijing Hospital, Air Force Medical University, Xi'an, China

## Abstract

**Objectives:**

The purpose of this study was to review the experiences with transcatheter closure of mitral PVL after surgical valve replacement.

**Background:**

Transcatheter closure of paravalvular leak (PVL) is an intricate alternative to surgical closure. But it represents one of the most intricate procedures in the field of structural heart interventions, especially for patients with mitral PVL.

**Methods:**

From January 2015 through January 2019, 35 patients with mitral PVL after valve replacement underwent transcatheter closure. We reviewed the catheter techniques, perioperative characteristics, and prognosis. The median follow-up was 26 (3–48) months.

**Results:**

Acute procedural success was achieved in 33/35 (94.3%) patients. Twenty-five patients had single mitral prosthetic valve replacements; 10 had combined aortic and mitral prosthetic valve replacements previously; 28 had mechanical valves; and 7 had bioprosthetic valves. All percutaneous procedures were performed with local anesthesia except for seven transapical cases with general anesthesia. Multiple approaches were used: transfemoral, transapical, and transseptal via an arteriovenous loop. Multiple devices were deployed. There were no hospital deaths. The procedural time was 67–300 (124 ± 62) minutes. Fluoroscopic time was 17–50 (23.6 ± 12.1) minutes. The hospital stay was 5–17 (8.3 ± 3.2) days. Complications included recurrent hemolysis, residual regurgitation, acute renal insufficiency, and anemia. Twenty-seven (77.1%) patients improved by ≥1 New York Heart Association functional class at the 1-year follow-up.

**Conclusions:**

Transcatheter mitral PVL closure requires complex catheter techniques. However, this minimally invasive treatment could provide reliable outcomes and shorter hospital stays in selected patients. This trial is registered with NCT02917980.

## 1. Introduction

Paravalvular leak (PVL) is a common complication after surgical valve replacement, with an incidence of 0.5%–7% in the aortic and 5%–10% in the mitral position [1–4]. Among patients with PVL, approximately 3% require treatment because of congestive heart failure or hemolytic anemia [5–8]. Surgery with repair or re-replacement was the classical treatment for PVL. Recently, transcatheter closure of PVL has emerged as an alternative treatment for patients with a high surgical risk [9–12].

However, transcatheter closure of PVL is one of the most challenging structural heart disease interventions, depending largely on the location and size of the defect, especially for patients with mitral PVL. Complex catheter techniques are needed for mitral PVL closure because the physicians must cross the PVL defect and deliver the occluder, which is difficult in most cases. Therefore, the reported success rate of mitral PVL closure remains from 62% to 86% in published series [13–16].

In our experience, multiple approaches, depending on the location and size of the defect, could improve the success rate. This retrospective study presents the perioperative outcomes and midterm follow-up results of transcatheter closure of mitral PVL.

## 2. Methods

### 2.1. Patient Population

The study protocol was approved by the ethics committee of Xijing Hospital (Approval Number: KY20150205-1) and registered in the ClinicalTrials.gov Protocol Registration System (NCT02917980). Between January 2015 and December 2019, 35 patients with mitral PVL after surgical valve replacement underwent transcatheter closure at five cardiac centres in China (Xijing Hospital, Anzhen Hospital, First Hospital of Zhengzhou University, First Affiliated Hospital of Harbin Medical University, and Hanzhong Central Hospital, China). All 35 patients or guardians of patients provided informed consent to participate in the study, and all clinical documents were reviewed for analysis.

A total of 25 patients had single mitral prosthetic valve replacements, and 10 patients had previously combined aortic and mitral prosthetic valve replacements. Twenty-eight patients had mechanical valves, and 7 patients had bioprosthetic valves. The patients were advised of the procedural risks and options as well as of the off-label use of all closure devices. Patient demographics and medical histories are shown in Table 1.

### 2.2. Procedure

**Figure 1:**
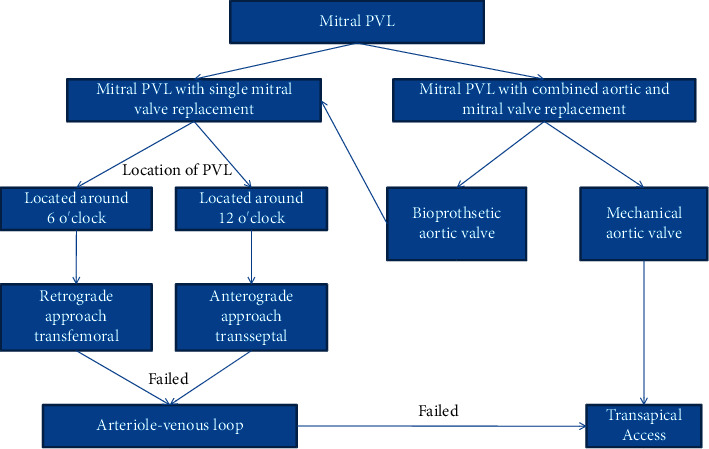
Protocol strategy for the different procedures.

All transcatheter procedures were performed in the catheterisation laboratory. The location of the PVL and the volume of regurgitation were confirmed by 3-dimensional transesophageal echocardiography (TEE) or transthoracic echocardiography (TTE) before the procedures and by computed tomography angiography in some selected patients. Seven mitral PVL closures were performed via the transapical approach with the patient under general anesthesia. All other procedures were performed with the patient under local anesthesia. Multiple approaches were performed, including transfemoral, transapical, and transseptal, via an arteriovenous loop according to the anatomy, the location of the PVL, and previous operation(s) (Figure 1).

24 patients underwent CT scanning and made individual 3D printing models. These patients had complications such as valvular disease, or the anatomical structure was complex. 3D printing models were made in order to get a better understanding of the situation of perivalvular leakage.

**Figure 2:**
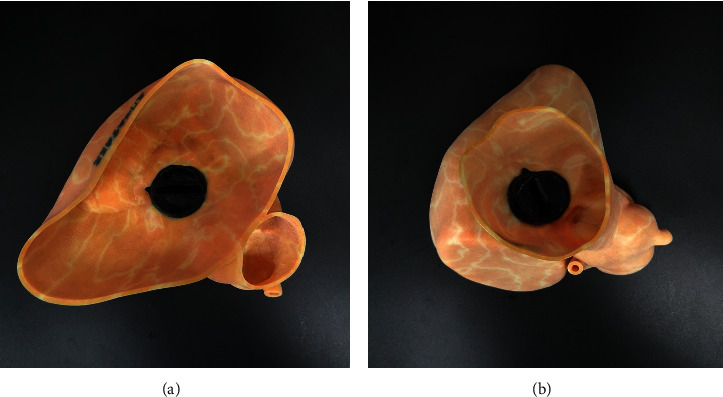
A 3D printing model clearly shows the location and surrounding structures of mitral paravalvular leakage.

3D printing models of the anatomical structure of the perivalvular leakage were reconstructed according to the preoperative CT results, which assisted the operator more intuitively to observe the location and shape of the perivalvular leakage. The operator can better simulate the operation and determine the operation plan (Figure 2).

### 2.3. Retrograde Transfemoral Approach

**Figure 3:**
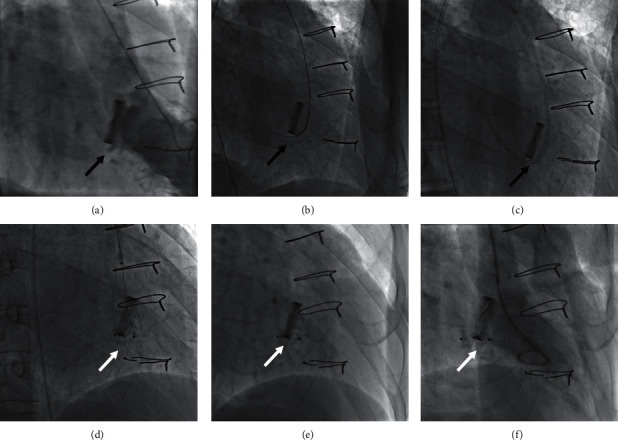
Angiogram taken during the transcatheter procedure of mitral PVL closure via a transfemoral retrograde approach. (a) Left ventricular angiogram to profile the paramitral regurgitation. (b) Retrograde crossing of the paravalvular leak with the guidewire. (c) The introducer sheath was advanced into the left atrium. (d) The occluder device was placed at the position of the paravalvular leak. (e) The occluder device was deployed. (f) Left ventricular angiogram after deployment. The black arrow indicates the PVL. The white arrow indicates the occluder.

The retrograde transfemoral approach is used to be the first-line approach for all mitral PVLs in our cohort. In later cases, it was used only for PVLs located at approximately 6 o'clock on the mitral valve. During the procedure, paramitral regurgitation and the location of the defects were confirmed with a left ventricular angiogram after a 6 Fr pigtail catheter was placed in the left ventricle via femoral arterial access. Then, a 5 Fr multipurpose diagnostic catheter and a 260 cm (0.032 inches) straight-tip wire (Cook, Bloomington, IN, USA) were advanced through the defects under the guidance of angiography. An extrastiff, 0.035-inch exchange-length Lunderquist guidewire (COOK Medical, Bjaeverskov, Denmark) was placed through the aortic valve and the paramitral defects into the left atrium, followed by placement of a larger sheath over the guidewire. Then, an appropriate Amplatzer-type occluder was deployed (Figure 3).

### 2.4. Anterograde Transseptal Approach

**Figure 4:**
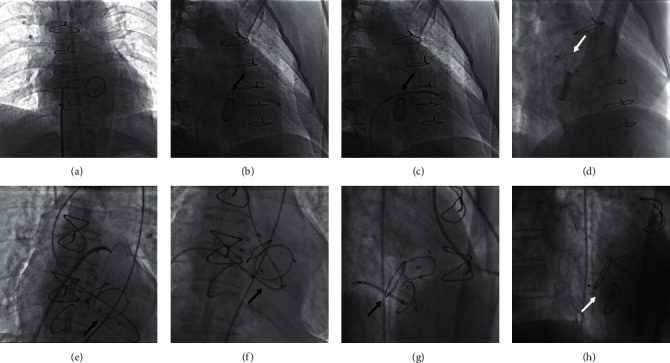
Angiogram taken during the transcatheter closure of the mitral PVL closure via an anterograde transseptal approach and arteriovenous wire loop approach. (a) The femoral venous access was followed by a transseptal puncture. (b) The introducer sheath was advanced into the left ventricle from the femoral vein. (c) The occluder was delivered into the left ventricle. (d) The occluder was deployed. (e) The transseptal puncture was followed by snaring the wire for setting up the arteriovenous loop. (f) The introducer sheath was advanced into the left ventricle via the arteriovenous loop. (g) The occluder was partially placed at the position of the paravalvular leak. (h) The occluder was deployed. The black arrow shows the paravalvular leak. The white arrow shows the occluder.

The anterograde transseptal approach was chosen if the PVL was located at about 12 o'clock of the mitral valve because it could be difficult to cross the PVL defect with the delivery sheath via the retrograde transfemoral approach. During the procedure, femoral venous access was performed followed by a transseptal puncture. A lower rather than a higher transseptal puncture is preferred. Then, a steerable sheath, such as the Agilis™ sheath (St. Jude Medical, St. Paul, MN, USA), was advanced into the left atrium to navigate the wire in front of the defect. Next, a 5 Fr multipurpose diagnostic catheter and a 260 cm (0.032 inches) straight-tip wire were advanced through the PVL defects. The straight wire was exchanged for an extrastiff 0.035-inch exchange-length Lunderquist guidewire (COOK Medical, Bjaeverskov, Denmark). The delivery sheath was then advanced through the defect over the support wire. Finally, the appropriate Amplatzer-type occluders were deployed (Figures 4(a)–4(d)).

### 2.5. Arteriovenous Loop

In some patients, it was difficult to advance the delivery sheath or the support wire through the PVL defect even though the catheter and guidewire had already been advanced through the defect. Then, the arteriovenous loops were needed to advance the delivery sheath. In these cases, both a transseptal puncture and femoral artery access were performed. During the procedure, the PVL was first crossed via the retrograde transfemoral approach using a superslippery straight wire (Terumo Corp., Tokyo, Japan) from the left ventricle to the left atrium. The wire was placed into the left/right pulmonary vein. The femoral venous access was performed followed by the transseptal puncture. Then, the introducer sheath was advanced into the left atrium to capture the superslippery straight wire in the left/right pulmonary vein by a gooseneck snare (AGA Medical Corp., Plymouth, MN, USA). An arteriovenous loop was formed, along which the introducer sheath was advanced into the left ventricle. Then, the appropriate Amplatzer-type occluders were selected and deployed (Figures 4(e)–4(h)).

### 2.6. Transapical Approach

#### 2.6.1. Minimally Invasive Transapical Approach

**Figure 5:**
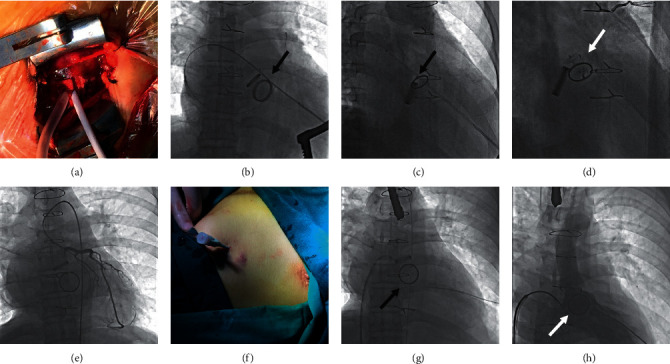
Closure of a mitral paravalvular leak (PVL) via the transapical approach. (a–d) The transcatheter closure of the mitral PVL via a minimally invasive transapical approach. (a) The transapical accesses were obtained by placement of a 6 Fr sheath. (b) The mitral PVL was crossed retrogradely. (c) The introducer sheath was advanced into the left atrium. (d) The occluder was deployed. (e–h) Transcatheter closure of the mitral PVL via a transapical puncture and arteriovenous loop. (e) Coronary angiogram to confirm the location of the left anterior descending artery. (f) A 5 Fr sheath was placed into the left ventricle percutaneously. (g) The transseptal puncture was followed by snaring the wire to establish an arteriovenous loop. (h) The occluder was placed at the position of the PVL followed by a left ventricular angiogram. The black arrow indicates the PVL. The white arrow indicates the occluder.

For patients with combined mechanical aortic and mitral valve replacement, closure of mitral PVLs was not safe via the retrograde approach or the arteriovenous wire loop approach because passing a catheter through the mechanical aortic valve could affect its function and lead to severe hemodynamic deterioration. Transapical access is a safe alternative for these patients. Four patients had transapical PVL closure via a left thoracic minimally invasive incision. With the patient under general anesthesia, we performed a left minithoracotomy with apical cardiac exposure. Transapical access was obtained with a 6 Fr sheath placed at the apex after inserting purse-string sutures with pledgets in the standard fashion. We passed a 5 Fr multipurpose diagnostic catheter and a 260 cm (0.032 inches) Terumo wire through the 6 Fr sheath under the guidance of fluoroscopy and TTE, after confirming paramitral regurgitation and the location of the defects on the left ventricular angiogram. Then, a 6 Fr short sheath was exchanged for a relatively larger sheath; appropriate Amplatzer occluders were selected and deployed accordingly. The devices were released after we viewed the left ventricular angiogram. Then, the sheaths were removed, and the left ventricular apex was closed with purse-string sutures (Figures 5(a)–5(d)).

### 2.7. Percutaneous Transapical Puncture Approach

The percutaneous transapical puncture is an alternative to access via a left minithoracotomy. Three patients had a transapical puncture procedure without thoracotomy. During the procedures, coronary arterial angiography was performed to confirm the location of the left anterior descending artery. Then, the apex was punctured into the left ventricle, at the appropriate position away from the left anterior descending artery through the fifth/sixth intercostal spaces. Then, a 5 Fr sheath was exchanged and placed into the left ventricle percutaneously. A 5 Fr multipurpose diagnostic catheter and a 260 cm (0.032 inches) Terumo wire were passed through the mitral PVL. The delivery sheath needed to be advanced transseptally to avoid invasive transapical access. Therefore, we performed a transseptal puncture and then snared the wire in the left atrium. The arteriovenous wire loop was established between the apex and the femoral vein. The delivery sheath was advanced through the defect over the support arteriovenous wire loop from the femoral vein. Then, we deployed the appropriate Amplatzer occluders (AGA Medical Corp., Plymouth, MN, USA). The transapical access could be closed with an Amplatzer Duct Occluder type II (ADO II) device or a pressure dressing after the procedure (Figures 5(e)–5(h)).

Because devices designed for percutaneous closure of PVLs were not available in China, all Amplatzer occluders used in this study were used off-label for PVL closure, including the Amplatzer atrial septal occluder, the Amplatzer muscular ventricular septal defect occluder, the ADO, and Amplatzer vascular plugs (AVP II) (AGA Medical Corp., Plymouth, MN, USA). Multiple devices may be used.

### 2.8. Perioperative Outcome and Follow-Up

All clinical files were reviewed, and perioperative characteristics were documented, including procedural time, fluoroscopic time, blood transfusions, perioperative laboratory blood tests, and postoperative hospital stay. All patients were seen in the clinic to ascertain their clinical status (New York Heart Association functional class) and adverse events after discharge. Transthoracic echocardiography was performed to evaluate the improvements in the construction and function of the patients' hearts at 3 months, 6 months, and 12 months after the procedure. Computed tomography angiography was also performed during the follow-up period.

### 2.9. Statistical Analysis

Statistical analysis was conducted with SPSS 22.0 software (IBM SPSS Statistics for Macintosh, Version 22.0. IBM Corp, Armonk, NY, USA). Continuous variables are presented as means ± SD, and categorical variables are expressed as percentages. Univariable comparisons have been performed with the Student unpaired *t*-test for continuous normally distributed data and the chi-square test for categorical data. Values of *P* < 0.05 were considered statistically significant.

## 3. Results

### 3.1. Procedural and In-Hospital Outcomes

The procedural success rate was 94.3% in 33/35 patients who underwent percutaneous closure of a mitral PVL. Multiple devices were used to close the PVL, including patent ductus arteriosus occluders, muscular ventricular septal defect occluders, and AVP II occluders. The procedural characteristics are shown in Tables 2 and 3. In 2 patients, a 20 mm AVP II or an ADO occluder was deployed at the defect. However, the occluder could not be stabilised at the defect and could be easily pulled back into the aorta or the left atrium in a push-pull test; then, the procedure was terminated and the patient had open surgery later. There were no hospital deaths.

In this study, the volume of PVL regurgitation was decreased to mild and moderate-mild immediately after the procedure in all patients who were treated successfully. Three patients had hemolysis after the procedure. Of these, 2 patients had acute renal insufficiency and needed continuous renal replacement therapy and blood transfusions. All of these patients recovered before discharge from the hospital. Other complications included 2 femoral pseudoaneurysms and 1 hemothorax after the transapical approach. All of these patients recovered before discharge from the hospital.

### 3.2. Follow-Up

**Figure 6:**
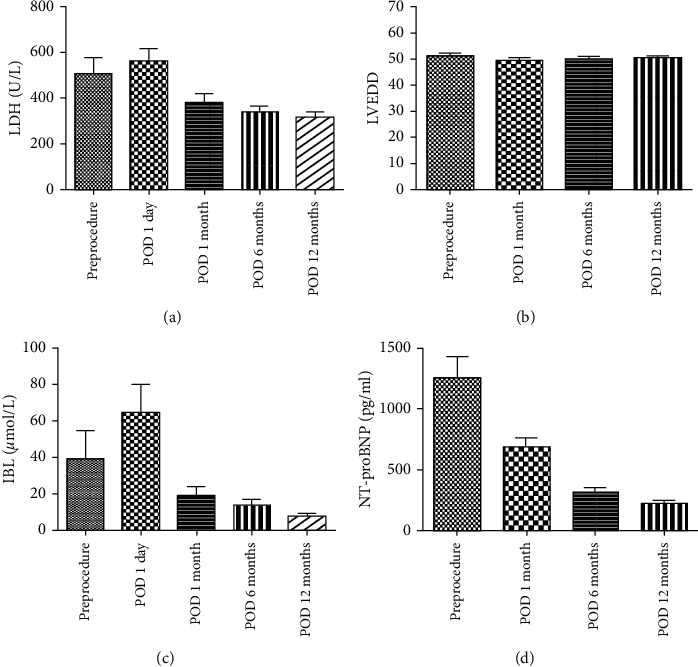
The 1-year follow-up results.

**Figure 7:**
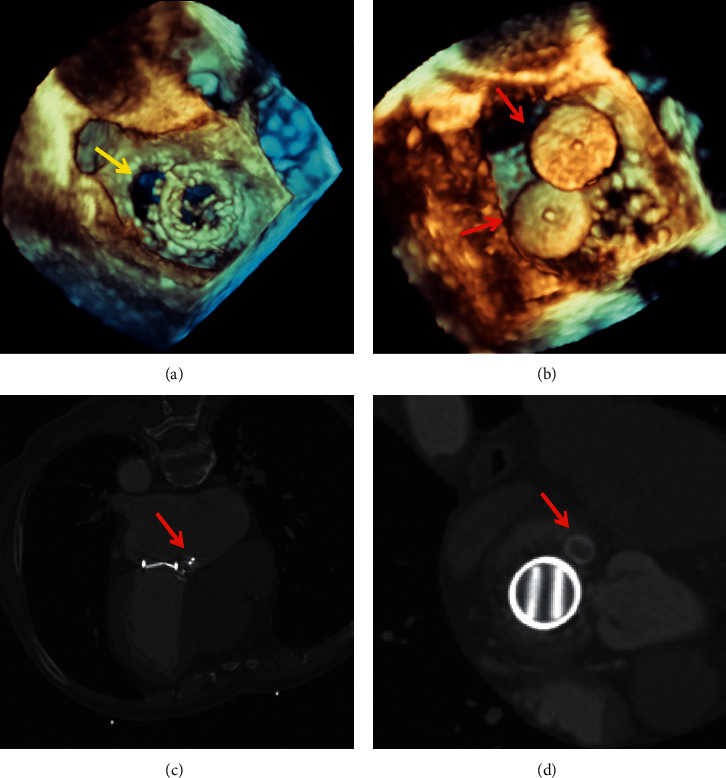
The 3-dimensional echocardiograms and computed tomography angiograms taken before the procedure and during the follow-up period. (a) Three-dimensional transesophageal echocardiography shows the mitral paravalvular leak (PVL) before the procedure. (b) Three-dimensional transesophageal echocardiography shows the mitral PVL closed with the occluder. (c) The mitral PVL was closed with the occluder (sagittal view). (d) The mitral PVL was closed with the occluder (axial view). The yellow arrow indicates the PVL. The red arrow indicates the occluder.

The median follow-up period was 26 (3–48) months, and follow-up was 100% complete. Twenty-seven (77.1%) patients improved by ≥1 New York Heart Association functional class at the 1-year follow-up visit. The left ventricular end-diastolic dimension showed no significant improvement. However, the levels of NT-proBNP returned to normal in most patients. The indirect bilirubin and LDH level decreased significantly after the procedure (Figure 6). Most patients no longer had mild to moderate paravalvular regurgitation during the follow-up examination with TEE, TTE, or CT angiography. TTE was used for patients under local anesthesia. TEE was used for patients under general anesthesia (Figure 7).

**Figure 8:**
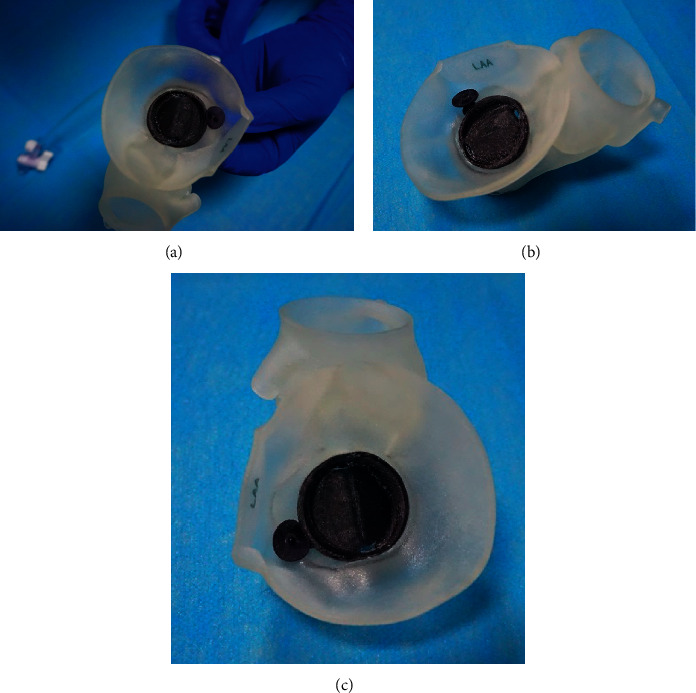
The 3D printing model of patients was made based on the postoperative CT results. The operator observed the position of the occluder device in vitro and evaluated the results of the operation.

The 3D printing model of patients was made based on the postoperative CT results. The operator has observed the position of the occluder device in vitro and evaluated the results of operation (Figure 8).

Two patients had recurrent hemoglobinuria in the first 2 months after discharge. One of them had severe anemia. The valve was re-replaced for this patient 2 months after discharge. The occluder interfered with the disk of the mechanical valve, which was diagnosed in the follow-up after the first procedure. This patient died of low cardiac output syndrome after open-heart surgery. The other patient recovered uneventfully in 2 months.

## 4. Discussion

PVL is a common complication after surgical valve replacement. Among patients with PVL, approximately 3% require treatment because of heart failure or hemolysis [16, 17]. Redo open-heart surgery to repair the PVL or valve re-replacement was traditionally the gold standard for patients with paravalvular regurgitation, but these procedures are accompanied by a high perioperative risk and a high recurrence rate [10]. Closure of the transcatheter PVL is a lower-risk alternative, with a 1% to 2% risk of periprocedural death or need for reoperation [18, 19]. However, this procedure is often very complicated and intricate, with a reported procedural success rate of about around 80%. In particular, the success rate drops to around 70% for mitral PVL [15, 19]. According to our prior research on 131 patients of PVL, we found that transcatheter closure was shown to be a safe, effective therapeutic option in patients with PVL. It was associated with a lower hospital mortality rate, shorter procedural time, and fewer blood transfusions than surgical treatment in selected patients [20]. In our research, with the application of multiple approaches and 3D printing models, we achieve a success rate of more than 90%.

Unlike an aortic PVL, which could be closed using less complicated techniques via the retrograde transfemoral approach, transcatheter closure of a mitral PVL can be technically challenging. Complex catheter techniques are needed for mitral PVL closure because crossing the PVL defect and delivering the occluder are difficult in most cases. Different access routes and catheter techniques might be used for mitral PVL closure, depending on the location and size of the defect. Moreover, there can be multiple defects, which increase the difficulty of the transcatheter intervention.

In this series, five different approaches were performed for mitral PVL closure. The first-line approaches varied with each patient. All previous surgical details were collected and analyzed before conducting the procedure, including which kind of prosthetic valve was implanted, whether it was a combined aortic valve replacement, and whether the atrial septal was sutured or not. The location, size, and structure of PVL were confirmed by TEE and/or CT angiography. The first-line approach was chosen on the basis of all these diagnostic details. We used the transfemoral approach if the patient had mitral valve replacement only. We used the retrograde transfemoral artery as the first-line approach if the mitral PVL was located at around 6 o'clock and the anterograde transseptal approach if the mitral PVL was located at around 12 o'clock. The delivery sheath was easy to advance via these approaches.

We preferred to use the snaring technique to set up an arteriovenous wire loop if advancing delivery sheath proved difficult by the transfemoral approach. It is necessary to establish an arteriovenous loop by snaring the wire and externalising it through the femoral artery or the left ventricle apex in some patients. Using this approach facilitated our ability to pass the sheath through the shallow angles and the calcified lesions. According to previous reports [1, 2, 21–23], these procedures are most often done with the patient under general anesthesia and under the guidance of TEE. In this study, however, all procedures were performed with the patient under local anesthesia and with TTE guidance except for 7 transapical cases. Based on our experiences, local anesthesia and TTE guidance can contribute to successful treatment without more invasive procedures and can save medical costs for most patients. However, a transseptal puncture can be a challenge in the patient with an atrial septal suture from a previous surgical procedure. TEE was necessary for the guidance of transseptal puncture in these patients.

For the patient with combined aortic and mitral valve replacement, one should consider whether a bioprosthetic or mechanical valve was implanted. The transfemoral approach could be performed for patients with a bioprosthetic aortic valve because it is safe to have a catheter crossing the aortic valve. However, passing a catheter through a mechanical aortic valve can affect the function of the mechanical valve and lead to severe hemodynamic deterioration. Transapical access is an important alternative for these patients. The shorter, more direct route makes the delivery by transapical access easier. Moreover, transapical access can also be performed for the patient with a single mitral valve replacement if advancing the delivery sheath was difficult by other approaches. Transapical access was also preferred by other physicians [11, 24, 25]. Taramasso reported satisfactory results in 17 patients who underwent mitral PVL closure through the transapical route. The 30-day mortality rate was 0%, with a procedural success rate of 94%. These results compared favorably with those from the open-heart surgery [11]. In another series of 43 patients by Ruiz, where transapical access was used for the majority of mitral PVLs, the technical success rate for device deployment in mitral PVLs was 89% [26]. There were totally 7 cases of the transapical approach in our study, and the success rate is 100%.

Moreover, transapical access can be achieved either by a surgical cutdown through a small anterolateral left thoracotomy or by direct percutaneous puncture of the left apex. In our study, 3 patients had a transapical puncture procedure without a thoracotomy. The arteriovenous wire loop was established between the apex and the femoral vein. The delivery sheath was then advanced through the defect over the support arteriovenous wire loop from the femoral vein. A 5 Fr sheath was placed at the apex, and the transapical access could be closed with an ADO II device or a pressure dressing only after the procedure was complete. Therefore, the transapical puncture procedure could also be safe. However, there is a potential risk of accidentally puncturing the left anterior descending coronary artery [27–29]. So, a selective coronary angiogram is necessary to guide the puncture.

The device used depends on the size and shape of the PVL. The purpose of the operation is to reduce or eliminate perivalvular regurgitation without affecting the function of leaflets. The anatomical characteristics of PVL and the nature of valve prosthesis may affect the choice of device. Structural features, such as the depth of the occluder and its position relative to the leaflet, may interfere with the function of the leaflet. As we know, AVP III and Occlutech were designed specially for PVL closure. However, these two devices were not commercially available in China. The devices used in this study including AVP II, ADO II, and PDA can also be used for PVL closure as off-label using device.

Clear evidence is lacking as to the optimal access route for a mitral PVL because of the lack of direct comparisons of the different approaches. In Taramasso's centre, the first-line option is the transseptal approach [11]. The retrograde arterial approach has almost been abandoned in some centres [30]. In others, transapical access is preferred for most patients with mitral PVLs [17, 30]. However, we think that the choice of the best approach must be individualized according to the specific situation of patients. The location of the defects was the most important determinant, which had been shown in Figure 1. The retrograde approach was the most commonly used approach in our study. It is the least invasive method without the need of heart apex cut down or transseptal puncture, and all the procedures can be conducted under local anesthesia, thus reducing the incidence of perioperative complications. Besides, 6 o'clock is one of the most predilection sites of mitral PVL; we have accumulated considerable experience regarding the retrograde approach and satisfactory results can be achieved through it for most of our clinical cases. In general, the success rate increases if multiple approaches are used.

## 5. Limitations

The present series is a retrospective, nonrandomised study in selected centres with its inherent limitations. The relatively small number of patients did not allow us to find more convincing conclusions. Physicians need to go through a learning curve to become familiar with the technique of the transcatheter PVL closure. Third, the follow-up time was limited. In any case, further studies are necessary to evaluate the long-term results.

## 6. Conclusions

Transcatheter mitral PVL closure requires complex catheter techniques. Based on the experiences of multiple centres, transcatheter closure of a mitral PVL is a safe, minimally invasive treatment with reliable in-hospital and short-to-midterm outcomes in selected patients.

## Figures and Tables

**Table 1:** Preoperative demographic and clinical characteristics.

Variables	Patients (*n* = 35)
Gender, male	24 (68.6%)

Age, years	27–70 (47.6 ± 12.7)

*Previous procedure*	
Mitral valve replacement	6
Combined aortic and mitral valve replacement	4
Mitral valve replacement and tricuspid valve repair	12
Combined aortic and mitral valve replacement and tricuspid valve repair	6
Mitral valve replacement and AF ablation	5
Mitral valve replacement and CABG	1
Mitral valve replacement and VSD repair	1

*Prosthesis type*	
Mechanical	28 (80%)
Bioprosthetic	7 (20%)
Time since valve replacement, years	0.5–14 (4.9 ± 3.6)
History of endocarditis	6 (17.1%)
Hemolysis	17 (48.6%)
NYHA FC II	2 (5.7%)
NYHA FC III	17 (48.6%)
NYHA FC IV	16 (45.7%)

*LVEF*	
<40	7 (20%)
40–50	15 (42.9%)
>50	13 (37.1%)

*PVL severity*	
Mild	0
Moderate	5 (14.3%)
Moderate to severe	17 (48.6%)
Severe	13 (37.1%)

*Comorbidities*	
Pulmonary hypertension	15 (42.9%)
Systemic hypertension	4 (11.4%)
Atrial fibrillation	27 (77.1%)
Chronic renal insufficiency, creatinine >1.5 mg/dL	4 (11.4%)

*EuroSCORE II*	
0–2	2 (5.7%)
3–5	17 (48.6%)
>6	16 (45.7%)

Categorical variables are presented as frequency (%); continuous variables are presented as mean ± standard deviation when normally distributed. The degree of paravalvular regurgitation was graded semiquantitatively using Doppler echocardiography and color-flow imaging (mild: <5 ml; moderate: 5–8 ml; moderate to severe: 8–12 ml; and severe: >12 ml). When multiple jets were present, the amounts of regurgitation from the separate jets were totaled for semiquantitation. AF: atrial fibrillation; CABG: coronary artery bypass graft; VSD: ventricular septal defect; NYHA FC: New York Heart Association functional class; LVEF: left ventricular ejection fraction; PVL: paravalvular leak.

**Table 2:** Procedural characteristics.

Total patients	35
Acute successful procedures	33 (94.3%)

*Approach*	
Transfemoral	14
Transseptal	1
Transseptal A-V loop	13
Transapical	4
Transseptal and transapical A-V loop	3

*Devices*	
PDA occluder	16
ADO II	3
VSD occluder	3
AVP II occluder	21
Single device	27
Two devices	8
General anesthesia	7
Local anesthesia	28

Fluoroscopic time (min)	17–50 (23.6 ± 12.1)
Procedural time (min)	67–300 (124 ± 62)
Hospital stay (days)	5–17 (8.3 ± 3.2)
Patients needing blood transfusions	4 (11.4%)

A-V: arteriovenous; PDA: patent ductus arteriosus; ADO : Amplatzer duct occluder; VSD: ventricular septal defect; AVP : Amplatzer vascular plug.

**Table 3:** Comparison between different approaches.

	Retrograde transfemoral (*n* = 14)	Anterograde transseptal (*n* = 1)	Transseptal A-V loop (*n* = 13)	Transapical (*n* = 4)	Transseptal and transapical A-V loop (*n* = 3)	*P*
Size of defects (mm)	8.6	8.0	8.3	7.8	8.8	>0.01
Size of occluders (mm)	11.2	10.0	10.6	10.4	11.5	>0.01
Procedure time (min)	98	82	138	156	188	<0.01
Success rate (%)	92.9	100	92.3	100	100	>0.01

A-V: arteriovenous.

## Data Availability

The data supporting the results in the current study are available from the corresponding author upon reasonable request.

## Abbreviations

**PVL**: Paravalvular leak
**TEE**: Three-dimensional transesophageal echocardiography
**TTE**: Transthoracic echocardiography
**ADO II**: Amplatzer duct occluder type II.
